# Comorbidity and health-related quality of life in Somali women living in Sweden

**DOI:** 10.1080/02813432.2019.1608043

**Published:** 2019-05-06

**Authors:** Taye Demeke, Amra Osmancevic, Martin Gillstedt, Anne Lene Krogstad, Eva Angesjö, Håkan Sinclair, Gamal Abd El-Gawad, Emily Krantz, Penelope Trimpou, Kerstin Landin-Wilhelmsen

**Affiliations:** a Angered Primary Health Care Centre, Gothenburg, Sweden;; b Department of Dermatology and Venereology, Institute of Clinical Sciences, Sahlgrenska Academy, University of Gothenburg, Gothenburg, Sweden;; c Brämhult Primary Health Care Centre, Borås, Sweden;; d Department of Geriatric Medicine, South Älvsborg Hospital, Borås, Sweden;; e Cleopatra Medical Centre, Gothenburg, Sweden;; f Department of Medicine, South Älvsborg Hospital, Borås, Sweden;; g Section for Endocrinology, Institution of Medicine, Sahlgrenska University Hospital at Sahlgrenska Academy University of Gothenburg, Gothenburg, Sweden

**Keywords:** Immigrants, women, vitamin D deficiency, comorbidity, Health-Related Quality of Life

## Abstract

**Objective:** To explore the relationship between low serum vitamin D levels and comorbidity in Somali women, immigrants to Sweden.

**Design and setting:** Cohort study in a Primary Health Care Center and a University Hospital.

**Subjects:** Somali women skin type V, n = 114, aged 18–56 years, from latitude 0–10^○^ N, living in Sweden, latitude 57^○^ N > 2 years were compared with women from a population sample, skin type II-III, n = 69, aged 38–56 years, the WHO MONICA study, Gothenburg, Sweden.

**Main outcome measures:** Serum (S)-25(OH)D, S-parathyroid hormone (PTH), comorbidity and Health-Related Quality of Life (HRQoL) using the Short Form-36 (SF-36) and part of the EQ-5D questionnaires. All calculations were corrected for age.

**Results:** Vitamin D deficiency (S-25(OH)D < 25 nmol/l) was found in 73% of the Somali women and in 1% of the controls (*p* < .0001). S-PTH was elevated (>6.9 pmol/l) in 26% and 9%, respectively (*p* < .004). Somali women used less medication, 16% vs. 55%, *p* < .0001) but more allergy medication, 11% vs. 7% (*p* = .006), had fewer fractures, 2% vs. 28% (*p* < .0001) and lower HRQoL in 7 out of 9 scales (*p* < .05–.001), than native controls. There were no differences in the prevalence of diabetes mellitus, hypothyroidism, positive thyroid peroxidase antibodies, vitamin B12 deficiency, celiac disease or hypertension.

**Conclusions:** Vitamin D deficiency was common in Somali women living in Sweden, 73%, but comorbidity was low. Both mental, and especially physical HRQoL scores were lower in the Somali women. The effects of long-lasting deficiency are unknown.

**Key points**
- The aim was to explore the relationship between vitamin D deficiency (S-25(OH)D < 25 nmol/l) and comorbidity in immigrants.
- Vitamin D deficiency was common in Somali women living in Sweden, 73%, but comorbidity of hypothyroidism, diabetes mellitus, hypertension, fractures and use of medications was low.
- Both mental, and especially physical, Health-Related Quality of Life were lower in the Somali women than in native Swedish women.
- The effects of long-lasting deficiency are unknown.

## Introduction

The involvement of vitamin D in metabolic bone diseases is well established and documented [1]. Severe depletion of vitamin D causes defectively mineralized bone matrix, termed osteomalacia [1,2].

The clinical manifestations of osteomalacia are bone pain, tenderness and muscle weakness [3,4]. Beyond its classical effects on calcium and bone homeostasis, vitamin D is also recognized for its immunomodulatory, pro-differentiation and anti-proliferative biological activity [5]. Genetic and environmental factors are involved in the development of autoimmune disease [6]. The increasing frequency of autoimmune diseases as one departs northward from the equator may be linked to the lower exposition of ultraviolet B (UVB). Such associations have been found in immigrants in Italy who developed allergy and asthma after arrival [7]. Sunlight is vital to maintain normal vitamin D levels [8,9]. There are studies showing the role of vitamin D in reducing the risk of chronic illnesses [10] including autoimmune diseases [6,11].

Vitamin D deficiency can be a result of malabsorption. Celiac disease is associated with other insufficiencies, like vitamin D, cobalamine, folacin and iron deficiency, as a result of increased intestinal tract immune activation affecting normal digestive and absorptive functions [12].

An association has been found between elevated anti-thyroid antibodies (TPOAb) in patients with autoimmune hypothyroidism and vitamin D deficiency [13,14]. These patients exhibited significantly lower vitamin D levels and markedly higher parathyroid hormone (PTH) levels, compared to controls [13,14]. The role of vitamin D in diabetes mellitus type 2, its development and progress, is still not conclusive [15].

Chronic pain was associated with impaired Health-Related Quality of Life (HRQoL) in immigrants in a review by Straube et al [16]. Somali women who have emigrated to Sweden, at a latitude poor in UVB light, had a high prevalence of vitamin D deficiency and low bone mineral content [17]. However, the implications of such a deficiency for the development of autoimmune and/or other diseases have not yet been thoroughly explored. Hypovitaminosis D and distorted phosphate metabolism cause osteomalacia, a defective bone mineralization, leading to generalized bone and muscle pain, weakness of muscles and cramps, which together affect HRQoL negatively. Many patients from Somalia come to the general practitioners due to these symptoms and the physicians must be aware that vitamin D deficiency is a possible explanatory factor. Supplementation should be given accordingly [18].

The aim of this study was to assess vitamin D status in relation to comorbidities in a group of Somali immigrant women with a Swedish population comparison group of women. Furthermore, the purpose was to control for other possible etiologies of vitamin D deficiency such as autoimmune disorders and gastrointestinal malabsorption. We hypothesized that Somali women with vitamin D deficiency; i.e., a serum (S)-25(OH)D level <25 nmol/l, and living in Sweden, would have more pain, as an indicator of possible osteomalacia, lower HRQoL, more autoimmune disorders, such as celiac disease, hypothyroidism, vitamin B12 deficiency, allergy and diabetes mellitus, hypertension, fractures and more use of medications in general as a proxy for morbidity.

## Subjects and methods

### Somali women

Somali women, *n* = 114, aged 18–56 years, with skin type V, who had migrated from latitude 0–10° North (N), and having lived in Sweden, latitude 57° N, for at least 2 years, were recruited during the autumn-winter 2010–2011 on a voluntary basis to participate in a vitamin D treatment trial [18]. The range of their stay in Sweden was 2–23 years, mean 15.1 ± 6.4 and median 18 years. Flyers written in Somali were distributed to Somali community shops, Somali associations, a nearby pharmacy and a supermarket. Somali women interested in the study were all included after health checkups by the experienced physicians, authors EA, TD and AO, according to the eligibility criteria applied. Exclusion comprised pregnancy, lactation, travelling to sunny countries in the last 2 months or supplement intake which could influence on the vitamin D levels. All participants lived in the north-east area of the city center of Gothenburg and in Borås, in Western Sweden. It is estimated that around 4000 Somalis reside in Gothenburg. All, but one of the women were premenopausal. Eighty-two of the 114 Somali women (72%) filled in the HRQoL questionnaires. Those who did not respond to the questionnaire (28%) were unwilling to participate but no reason was given. The demographic data are presented in Table 1.

**Table 1.** Characteristics of the Somali women, *n* = 114, living in Sweden, and native Swedish women, *n* = 69 as comparison group: other diseases, medication, laboratory data and Health-Related Quality of Life measured using Short Form-36 and EQ-5D. Means ± SD and percentages (%) are given.

	Somali women *N =* 114	Comparison group *N =* 69	*p*-level	*p*-level adjusted for age
Age, years	34 ± 9 (18–56)	49 ± 6 (38–56)	<.001	–
Body weight, kg	74 ± 14	70 ± 12	.03	.002
Height, cm	164 ± 7	166 ± 7	.08	.03
Body mass index, kg/m^2^	27 ± 5	26 ± 4	.006	<.0001
S-Calcium, mmol/l	2.32 ± 0.09	2.30 ± 0.09	.30	.74
S-Calcium <2.15 mmol/l, *n* (%)	0 (0)	1 (2)	.38	1
S-25(OH)D, nmol/l	23 ± 13	57 ± 19	<.0001	<.0001
S-25(OH)D < 25 nmol/l	83 (73)	1 (2)	<.0001	<.0001
S-PTH, pmol/l	5.9 ± 2.3	4.8± 1.6	.001	<.0001
S-PTH > 6.9 pmol/l, *n* (%)	30 (26)	6 (9)	.004	.004
S-DBP, ng/ml	204 ± 86	248 ± 88	.001	.005
S-vitamin B12 deficiency, *n* (%)	0 (0)	2 (3)	.14	.17
Hypothyroidism, *n* (%)	9 (8)	8 (12)	.44	1
Elevated TPOAb, *n* (%)	10 (9)	7 (10)	.80	.69
S-free T4, pmol/l	14.3 ± 2.5	16.0 ± 2.0	<.001	<.001
S-TSH, mU/l	1.9 ± 1.5	2.1 ± 1.2	.07	.99
Hypertension, *n* (%)	4 (4)	7 (10)	.11	.28
Diabetes mellitus, *n* (%)	2 (2)	0 (0)	.53	1
Fractures, *n* (%)	2 (2)	19 (28)	<.0001	<.0001
Any medication, *n* (%)	18 (16)	38 (55)	<.0001	<.0001
Calcium/vitamin D supplement, *n* (%)	0	0	1	1
Allergy medication, *n* (%)	13 (11)	5 (7)	.45	.006
Analgesics, *n* (%)	14 (12)	8 (11)	1	1
Psychopharmacological agents, *n* (%)	0	18 (26)	<.0001	.0001
Physical component score, low-high*	47 ± 9	49 ± 10	<.001	<.001
Mental component score, low-high*	47± 12	52 ± 11	.02	.17
Self-rated health, (0–100), low-high*	76 ± 22	81 ± 17	.0014	.01

PTH: Parathyroid hormone; DBP: Vitamin D-Binding Protein; TPOAb: Thyreoperoxidase antibodies; T4: Thyroxine. *n = 82/114 Somali women fulfilled the questionnaires.

### Comparison group

A random population sample of men and women (*n* = 2400, aged 25–64 years) was invited in 1995 in Gothenburg, Sweden (latitude 57**°** N), as a part of the third World Health Organisation (WHO) MONItoring of trends and determinants for CArdiovascular disease (MONICA) project, Gothenburg, Sweden [19]. The participation rate was 67% (*n* = 1616). Hormonal sampling was performed on every 4^th^ participating man and woman (randomly selected) aged 25–34, 35–44, 45–54, and 55–64 years. Women in the age groups 25–34 and 35–44 years with hormonal blood sampling, *n* = 95, were re-examined in autumn-winter-spring 2008–2009, *n* = 69 (participation rate 73%), aged 38–56 years, and were used as a comparison group. The non-responders were travelling, living abroad or had no time to attend the re-examination by the physicians and coauthors PT and KLW. All women had skin type II or III. Less than 2% were born outside Europe. HRQoL questionnaires were filled in by all 69 women in the comparison group (100%) who were examined by the same staff and in a similar manner as the examination of the Somali women.

### Anthropometry and biochemistry

Body weight and height were measured to the nearest 0.1 kg and 1 cm, respectively, in the fasting state in underwear and without shoes. BMI was calculated as body weight divided by the height squared (kg/m^2^). One and the same operator performed all measurements on both Somali women and the comparison group.

Venous blood samples were collected in the fasting state in the morning and on day 7–9 of the menstrual cycle. Blood samples were drawn from January to June and from September to December in approximately equal monthly proportions. The samples were stored in −80 degrees Celsius until analysis which was performed within one year for all subjects. S-25(OH)D was measured with a radioimmunoassay 125I RIA kit (DiaSorin, Stillwater, MN, USA). The total coefficient of variation was 12% for the mean level of 36 nmol/l, 15% for 57 nmol/l and 14% for 147 mmol/l. S-25(OH)D < 25 nmol/l was defined as deficiency and ≥25 < 50 nmol/l as vitamin D insufficiency, according to the Institute of Medicine (IOM), the National Institute of Health, Washington DC, USA [20]. S-PTH was determined by immunoradiometric assay (Roche Cobas, Rotkreutz, Switzerland). The reference range was 1.6–6.9 pmol/l. Vitamin D-Binding Protein (DBP) was assessed with an enzyme-linked immunosorbent assay (ELISA; Cat. No DVDBP0, R&D Systems, Minneapolis, MN, USA). Serum-free T4 and TSH were measured with the ECLIA Modular immunometric method (Roche, Rotcreutz, Switzerland).

Thyreoperoxidase antibodies (S-TPOAb) (positive >60 kU/l), vitamin B12 (deficiency <140 pmol/l) and calcium (S-Ca), all from serum, were analyzed using routine methods. The reference range for S-Ca was 2.15–2.50 mmol/l.

### Medical history, medication and quality of life

A complete medical history regarding previous diseases was taken by the examining physicians, authors AO, EA, TD, PT and KLW and the morbidity data recorded in the medical record. Current smoking and use of medication were asked for and coded in line with the Anatomical Therapeutic Chemical (ATC) Classification System.

The participants were asked about the presence of hypothyroidism, hypertension, diabetes mellitus and allergy, and about their regular use of medication related to the disorders. Use of levothyroxine or a S-TSH level >4.2 mU/l at the examination were defined as hypothyroidism. Medication for vitamin B12 deficiency and/or a low vitamin B12 level at the examination was defined as vitamin B12 deficiency. Inhalations and allergy medications were indicators for asthma and/or allergic problems. The use of painkillers (non-steroid anti-inflammatory and paracetamol) and psychopharmacological medications (sedatives and antidepressant drugs) as an indicator of impaired health and all medications in general, were recorded and compared between Somali women and the comparison group.

X-ray verified fractures of the wrist, hip, ankle, foot, rib and vertebrae were defined as possible osteoporotic or fragility fractures.

HRQoL was registered using the Medical Outcomes Study Short Form 36 questionnaire (SF-36) [21]. It yields an eight-scale profile of functional health and wellbeing scores for Physical Functioning = PF, Role limitations due to Physical health = RP, Bodily Pain = BP, General Health = GH, Vitality = VT, Social Functioning = SF, Role limitations due to Emotional problems = RE, and Mental Health = MH. It also generates two summary scores; the Physical Component Summary score (PCS) consisting of PF, RP, BP and GH and the Mental Component Summary score (MCS) which includes VT, SF, RE and MH. Scores range from 0–100 (low-high).

Self-rated global health status was also assessed using question #6 in the EuroQoL-5 dimensions (EQ-5D), score range 0–100, with 0 being the worst conceivable and 100 the best conceivable level [22].

## Statistical analysis

Means, standard deviations and medians were calculated with conventional methods. The data was analyzed using R, version 3.0.3 (The R Foundation for Statistical Computing, Vienna, Austria). Wilcoxon’s rank sum tests and the t-tests were used for two-sample comparisons. All tests were two-sided and *p* < .05 was considered statistically significant. A multiple linear regression model was used for adjustment of confounding factors as age and BMI. A power calculation regarding sample size was performed according to the Jonckheere-Terpstra test before the randomized clinical trial in the Somali women [18].

## Results

This cohort study compared Somali women, immigrants to Sweden with native Swedish women regarding vitamin D levels, possible comorbidity and HRQoL. Anthropometric and laboratory data and comorbidity are presented in Table 1. The Somali women were younger, had higher body weight and BMI and were shorter than women in the comparison group. A multiple linear regression analysis was performed including group (Somali women and comparison group), age and BMI as independent variables and S-25(OH)D as the dependent variable. Since only age was important, age-adjustment was performed and S-25 (OH) was lower in Somali women than in the comparison group, *p* < .0001. Vitamin D was positively correlated with age, *p* = .0025 and BMI did not significantly contribute to the model. Vitamin D deficiency (S-25(OH)D < 25 nmol/l) was found in 73% of the Somali women and in 1% of the population sample, Table 1. S-PTH was elevated (>6.9 pmol/l) in 26% and 9%, respectively. S-DBP was lower and S-PTH higher in the Somali women. One Somali woman, but none in the comparison group, had primary hyperparathyroidism. Diabetes mellitus was found in 2 (2%) of the Somali women; one had metformin-treated type 2 diabetes, and body weight 106 kg, BMI 36.7 kg/m^2^ and the other had insulin dependent type I diabetes, 82 kg and BMI 30.1 kg/m^2^. None had diabetes mellitus in the population sample (ns between groups). There were no differences in the frequency of hypothyroidism, positive TPOAb, hypertension or vitamin B12 deficiency between the groups, although S-free T4 was lower, but within the reference range, in the Somali women. None of the Somali women, and only one in the comparison group, had celiac disease according to their medical history (ns). Vitamin D deficiency was not more prevalent in women with hypothyroidism in either Somali women or in the comparison group from the population.

Fractures were less common in Somali women. Two wrist fractures had occurred in the oldest Somali women, respectively, one of whom was postmenopausal, 56 years. There were 7 wrist fractures, 3 of the upper arm, 3 of the lower arm, and 6 of the ankle, all of presumed low energy origin, in the comparison group. All fractures among the latter occurred in women above 40 years and 14/19, (74%), fractures in women above 50 years of age, and postmenopausal.

Somali women used more allergy medication but less sedative and antidepressant agents or any medications in general than the comparison group, Table 1. None of the Somali women smoked compared to 7% in the native Swedish women, *p* = .007.

**Figure 1.**
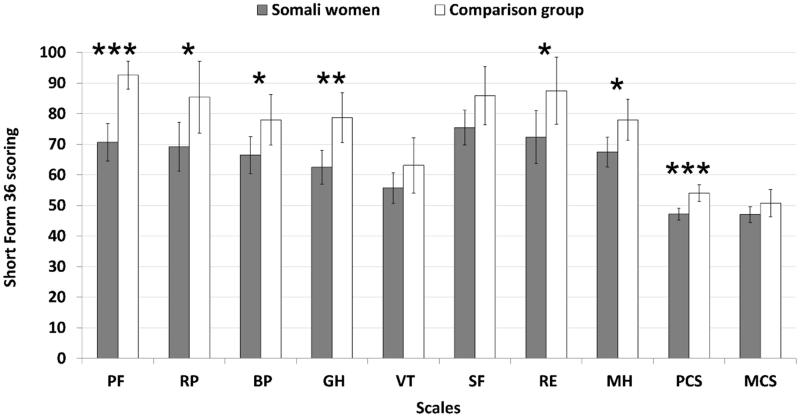
Health-Related Quality of Life measured using Short Form-36 in Somali women living in Sweden >2 years and women from the World Health Organization, MONItoring of trends and determinants for CArdiovascular disease (WHO MONICA) study, Gothenburg, Sweden as a comparison group. PF: Physical Function; RP: Role Physical; BP: Bodily Pain; GH: General Health; VT: Vitality; SF: Social Function; RE: Role Emotional; MH: Mental Health; PCS: Physical Component Summary score including PF, RP, BP and GH; MCS: Mental Component Summary score including VT, SF, RE and MH. Means ± SD are given. *p < .05; **p < .01; ***p < .001 for age-adjusted data.

HRQoL was lower in 6 of the 8 subscales in the SF-36, and especially the Physical Component Summary score (including PF, RP, BP and GH), and self-rated health in the EQ-5D scale in Somali women compared with comparison group. The mental scores RE and MH were lower, but there was no difference in the Mental Component Summary score, Figure 1 and Table 1. There was no correlation between HRQoL and the number of years the Somali women had lived in Sweden.

## Discussion

Vitamin D deficiency was common in Somali women, 73%, and consequently, secondary hyperparathyroidism was also more frequent in this group. No other comorbidity, except for more use of allergy agents, was more prevalent in Somali women living in Sweden for at least two, mean 15, years than in native Swedish women used for comparison. Both mental, and especially the physical HRQoL scores, were lower in Somali women.

One main hypothesis was that vitamin D deficiency may be associated with more pain as an indicator for possible osteomalacia [16,23,24]. Bodily pain was reported more frequently in Somali women than in native, slightly older, women, after age adjustment, in the present study. This supports the link between vitamin D deficiency and pain which was alleviated after vitamin D treatment in a previous report of female immigrants from Africa, Asia and South America in Sweden [23].

A recent study showed no association between S-25(OH)D levels and chronic low back pain in a native Swedish population sample [25]. In that study, all had sufficient levels of S-25(OH)D, mean 80 nmol/l. However, a study from Norway showed that both immigrants and native Norwegians had more pain when the S-25(OH)D levels were below 30 nmol/l [24]. This supports the hypothesis that vitamin D deficiency is a strong pathogenetic factor for the development of musculoskeletal pain, possibly due to osteomalacia, as in the present Somali women where 73% had S-25(OH)D < 25 nmol/l. The lower S-DBP levels in Somali women reflected a manifest vitamin D deficiency and the higher S-DBP in native Swedish women correlated positively with the S-25(OH)D levels in a previous report [26].

The association between vitamin D deficiency and autoimmune diseases could not be verified in the present controlled study. Choi et al. showed in over 6000 South Korean subjects that lower vitamin D status was associated with the presence of autoimmune thyroid disease [14]. Another study on Hungarians showed that the prevalence of vitamin D deficiency was higher in subjects with autoimmune hypothyroidism compared with subjects without hypothyroidism [27]. In the present study, there was no difference in the frequency of hypothyroidism according to levothyroxine supplementation rates or the prevalence of elevated S-TPOAb, although S-free T4 was lower in the Somali women than in the native Swedish women. However, the S-free T4 levels were well within the reference range. There was no increased frequency of vitamin D deficiency in hypothyroidism in either Somali women or native Swedish women.

The use of allergy agents was higher in Somali women than in native Swedish women. This corroborates another study in immigrants, mainly women, who developed asthma and allergy within four years of moving to Italy [7].

Tavakkoli et al. demonstrated that 25% of patients with celiac disease had vitamin D deficiency together with other autoimmune states [12]. None of the Somali women with vitamin D deficiency in the present study had a medical history, clinical signs, symptoms or other laboratory findings as low S-vitamin B12, S-folate or S-iron indicative of malabsorption. However, confirmatory gastroscopy was not performed and gliadin antibodies were not taken in Somali women.

The regulatory effects of vitamin D on hypertension and diabetes mellitus have been discussed [28], and vitamin D plays an important role in maintaining the function of the heart [10]. There was no difference in the use of antihypertensive or diabetic medication in the Somali women compared with the native women in the comparison group in the present study. Two Somali women had diabetes mellitus which could be explained by their higher body weight rather than the vitamin D deficiency *per se.*

The fracture frequency was lower in the Somali women than in the comparison group. This was in spite of the lower bone mineral density in the Somali women, measured with the dual energy x-ray absorptiometry in relation to the manufacturers´ age-matched women of both white American and black African-American referents reported previously [17]. The fractures occurred mainly in postmenopausal women in the Somalis and in the native Swedish women in the present study. Both groups were overweight. There is a possibility that the real effect on fractures of vitamin D deficiency in Somali women may appear after age 50. We did not evaluate the degree of physical activity or dietary habits in Somali women. Another study has shown that physical activities, especially during their leisure time, were almost nil in Somali women compared with ethnic Swedes [29]. This could contribute to a lower risk of falling and less fractures. Furthermore, women from the WHO MONICA study increased their degree of physical activity during leisure time and use of tranquilizers, during a 13-year follow-up along with an increased fracture incidence [30]. The higher degree of physical activity could lead to increased muscle mass and improved body balance. On the other hand, the risk of falling may increase depending on the type of activity in the comparison group. These contradictory life style factors, and age, in Somali women and native Swedish women may explain the higher fracture frequency in the latter women. The types of fractures resulted mainly from falling as no vertebral compression was observed. Hence, the vitamin D deficiency in Somali women did not seem to contribute to more fractures at the lower ages.

The present results with, above all, up to 30% poorer physical functioning (PF) and general health (GH) in HRQoL in Somali women, are in conformity with a small study of immigrants of various origins living in the neighboring country of Denmark [31]. The length of time living in Sweden was similar in that study (4–23 years) to the present study, 2–23 years. However, HRQoL was not reduced in a Swedish report where questionnaires were posted to immigrants from non-European countries [32]. The time lived in Sweden was less than one year in that study, which may have influenced the results. No data on vitamin D levels were given in the reports [31,32], and the dropout rate was high in the latter [32].

The non-responder rate was also fairly high in the Somali women in the present study, 28%, regarding the HRQoL questionnaires. It is not clear whether they were illiterate or had difficulties understanding the language or the questions. Many of them were reluctant to answer the questions at all. The Somali women could choose between a Swedish or a Somali questionnaire and most of them chose the Swedish version. However, the results from the more cumbersome, and potentially more difficult to understand, SF-36 were corroborated by the single item self-rated health item in EQ-5D, as seen in Table 1, indicating an agreement of their subjective reports. It cannot be excluded that the more use of psychopharmacological agents in the native Swedish women might have increased their HRQoL.

The initial hypothesis in the present study was that Somali women living in Sweden would have vitamin D deficiency which may have led to more diseases, more medication and pain, and lower HRQoL than women in the native population. It is not clear what the reason is for the difference in medication, with a lower use in Somali women. There may be cultural traditions or habits regarding the use of medications. The results of this study support the hypothesis that HRQoL, especially the physical component, was lower but the overall comorbidity was less pronounced than expected. Given that vitamin D deficiency is common among immigrants, including Somali women, vitamin D supplementation is recommended, as the effects of long-lasting vitamin D deficiency are unknown. The present study closes a knowledge gap regarding the high prevalence of vitamin D deficiency, possibly related to impaired well-being.

### Study limitations

One limitation of the present study was the small sample sizes regarding the end points for comorbidity as hypothyroidism, diabetes mellitus, celiac disease, hypertension, fractures and allergic problems. The size of the study groups may be under-powered for examining the different outcomes in this study. Furthermore, the recruitment of Somali women may be influenced by selection bias. They were volunteers recruited by flyers and not by registers. Some were excluded due to travelling to sunny countries the last 2 months in order not to influence the results from the previous clinical intervention trial with vitamin D [18]. The controls were older and adjustment for age was performed.

Another limitation was the assay for measuring S-25(OH)D. The liquid chromatography, LC/MS-MS, is the golden standard for vitamin D assessment and combines all vitamin D metabolites on the same time. The correlation between LC/MS-MS and the radioimmunoassay method used from DiaSorin was >90% according to DEQAS [33]. Samples for S-25(OH)D in Somali women and women in the comparison group of the present study were analyzed with the same method (DiaSorin) at the same laboratory within 2 years. Possible celiac diagnosis was not confirmed by gastroscopy. Tender points specifically used for identifying bone pain or bone biopsy, which is the golden standard for verification of a definitive diagnosis of osteomalacia, were not performed. The multiple testing should also be taken into account as a limitation.

### Study strength

A strength was that the study group was homogeneous with regard to ethnicity; they all came from Somalia and had a similar skin type. Another advantage was the use of a population based comparison group from the same region. Furthermore, similar methods for the biochemical analyses at the same laboratory were used and the same staff coordinated the Somali women and the comparison group.

## Conclusion

Vitamin D deficiency was common in Somali women living in Sweden, 73%, but comorbidity was low. Health-Related Quality of Life, especially the physical component, was lower among the Somali women than among the native Swedish women. It is essential to perform long-term follow-up studies in immigrants living at higher latitudes to identify the potential consequences of vitamin D deficiency. The pronounced, and very frequent, vitamin D deficiency in immigrant women from Somali living at higher latitudes should be treated according to existing guidelines in Sweden [34] and internationally [20]. Many patients from Somalia seek the help of general practitioners due to diffuse pain and muscle weakness and vitamin D deficiency must be kept in mind as an explanatory finding. At least 1600 IU vitamin D daily was needed to reach sufficient levels, S-25(OH)D ≥ 50 nmol/l, in Somali women [18].
